# Mental Health Status of the Elderly Chinese Population During COVID-19: An Online Cross-Sectional Study

**DOI:** 10.3389/fpsyt.2021.645938

**Published:** 2021-05-12

**Authors:** Rui Zhou, Hao Chen, Lin Zhu, Ying Chen, Boyan Chen, Ying Li, Zhi Chen, Haihong Zhu, Hongmei Wang

**Affiliations:** ^1^Department of Social Medicine of School of Public Health and Department of Pharmacy of the First Affiliated Hospital, Zhejiang University School of Medicine, Hangzhou, China; ^2^Shanghai Health Development Research Centre (Shanghai Medical Information Research Centre), Shanghai, China; ^3^Department of Public Health, Xi'an Medical University, Xi'an, China; ^4^State Key Laboratory for Diagnosis and Treatment of Infectious Diseases, The First Affiliated Hospital, Zhejiang University School of Medicine, Hangzhou, China

**Keywords:** COVID-19, mental health, influencing factors, elderly, China

## Abstract

**Background:** COVID-19 not only threatened the public's physical health but also brought unbearable psychological pressure, especially for those vulnerable groups like the elderly. However, studies on the psychological status of older adults during this public health emergency remained scant. This study aims to investigate the mental health status among the elderly Chinese population during COVID-19 pandemic and determine the influencing factors of psychological symptoms.

**Methods:** From February 19 to March 19, 2020, an online survey was administered to Chinese older adults using a convenience sampling method. Information on demographic data, health status and other epidemic related factors were collected. Specifically, the study defined the psychological status as five primary disorder–depression, neurasthenia, fear, anxiety, and hypochondria–which were assessed by the Psychological Questionnaire for Emergent Event of Public Health (PQEEPH). Standard descriptive statistics and multiple logistic regression analyses were conducted to analyze the data.

**Results:** Of 1,501 participants recruited from 31 provinces in China, 1,278 were valid for further analysis. Participants' scores on each sub-scale were described in median and interquartile [M(Q)]: depression [0.00 (0.33)], neurasthenia [0.00 (0.40)], fear [1.00 (0.83)], anxiety [0.00 (0.17)], hypochondria [0.00 (0.50)]. Chronic diseases (depression *p* = 0.001; neurasthenia *p* < 0.001; fear *p* = 0.023; anxiety *p* < 0.001; hypochondria *p* = 0.001) and the BMI index (depression *p* = 0.015; neurasthenia *p* = 0.046; fear *p* = 0.016; anxiety *p* = 0.015; hypochondria *p* = 0.013) had significant impacts on all of the five sub-scales. Specifically, the rural dwellers had a higher level of neurasthenia, fear, and hypochondria. Besides, education level (*p* = 0.035) and outbreak risk level (*p* = 0.004) had significant impacts on the depression. Higher household monthly income per capita (*p* = 0.031), and the community-level entry/exit control (*p* = 0.011) are factors against anxiety.

**Conclusions:** Most elderly residents reported mild negative emotions during COVID-19 and more attention should be paid to the recognition and alleviation of fear. Our findings also identified factors associated with the mental health status of the elderly, which is of practical significance in the design and implementation of psychological interventions for this vulnerable population during COVID-19 and future emerging diseases.

## Introduction

In late December 2019, atypical pneumonia caused by a novel coronavirus (SARS-CoV-2) was found in Wuhan, capital of Hubei province, China, and the disease quickly spread all over China and several overseas regions. On January 30, 2020, the World Health Organization declared a Public Health Emergency of International Concern (PHEIC) over the global outbreak of the novel coronavirus and formally named the disease COVID-19 (1). Due to the strong infectivity and the quick transmission speed, On March 11, WHO has upgraded the outbreak of COVID-19 as a pandemic (2). China has witnessed a rapid increase of COVID-19 confirmed cases from 202 of January 20, 2020 to 74,283 of February 19, 2020. Daily new diagnosed cases began decreasing gradually after February 19, 2020 and the epidemic has been largely under control since May with sporadic cases occasionally occurred (3). However, the international situation is still not optimistic. As of February 28, 2021, the disease has affected 220 countries and regions, causing over 113 million confirmed cases and over 2.5 million deaths worldwide (4).

There has been mounting evidence showing that outbreaks of infection could deteriorate individuals' mental health (5–7), and COVID-19 is not an exception. The threat of COVID-19 on physical health, the overflow of media coverage on COVID-19, and the unprecedented large-scale epidemic control measures taken by the Chinese government to curb the spread of COVID-19 (8), such as lockdown of the Wuhan city, strict management of working and living spaces, closure of schools and non-essential businesses, etc., all induced a considerable degree of negative emotions among the public (9–11).

Older adults are significantly more prone to infection, severe illness, and death of COVID-19 (12–14), besides, their daily routines can also be greatly affected by the epidemic and the control measures due to lack of knowledge on advanced technology, major impacts include drastically reduced social activities and contact with relatives and friends, and difficulty in getting basic living amenities or medical treatments (15). These put them at a greater risk of developing several mental disorders, such as panic (16), fear (17), depression (18), and anxiety (19). Historical researches during SARS have also found that the elderly population experienced more severe psychiatric symptoms (20), worse subjective well-being (21), and higher suicide deaths (22). Both China and the rest of the world are witnessing an ever-increasing elderly population. By the end of 2017, the number of people aged over 60 in China had already reached 241 million, accounting for 17.3% of China's total population (23). The prevalence of depressive symptoms in Chinese older adults reached 23.6% even in a normal period (24), and the situation could be worse during this unprecedented pandemic. Inferior psychological status can also be related to irrational behaviors (25), impeding the containment of the epidemic. Therefore, greater attention should be paid to the mitigation of older adults' negative emotions during COVID-19.

Although the Chinese government has launched a series of notifications to initiate a brunch of online psychological intervention programs to help the public deal with the urgent psychological crisis (26, 27), these online programs can hardly be utilized by older adults due to limited access to internet services and smartphones (28). Thus, more efforts should be made to design targeted psychological intervention strategies for the elderly, and understanding their psychological status during the epidemic and the influencing factors would be the first step.

However, among the copious amount of empirical studies that focus on the mental health of the general population (29), medical staff (30), patients (31), college students (32), and adolescents (33) during the outbreak of COVID-19, studies on the specific mental situation of the elderly population under the pandemic can hardly be found. Therefore, this study aimed to assess the psychological status among the elderly Chinese population during COVID-19 pandemic by quantifying their degree of depression, neurasthenia, fear, anxiety, and hypochondria and to analyze the potential risk factors associated with these symptoms.

## Methods

### Study Design and Participants

This web-based cross-sectional survey was conducted from February 19, 2020 to March 19, 2020, during which COVID-19 cumulative confirmed cases steadily increased and community-wide COVID-19 prevention activities were launched by local health authorities. We uploaded the electronic questionnaire onto the online survey platform, “Wenjuanxing.” We spread the links or “QR” codes nationwide to elderly people (age ≥ 60 years old) via popular social media platforms like “WeChat.” Participants with serious cognitive impairment and those who were confirmed or suspected COVID-19 cases were excluded. Returned surveys with incomplete information or logic problems were deemed as invalid. Responses from a total of 1,501 participants were received, 197 of which were invalid cases, together with 26 participants who reported having never heard about COVID-19, resulting in an effective sample size of 1,278 participants.

### Measurements

#### Dependent Variable

Psychological status was measured by the Psychological Questionnaire for Emergent Event of Public Health (PQEEPH) (34). This scale has been widely used to investigate individuals' psychological status during public health emergencies (35–37), adequate psychometric properties have been described and a Cronbach's alpha coefficient value of 0.92 was found in this study. The PQEEPH consists of 25 items and assesses 5 dimensions: depression, neurasthenia, fear, anxiety, and hypochondria. All items are rated on a four-point scale ranging from 0 (occasionally) to 3 (always). The total score value for each sub-scale ranging from 0 to 3 can be obtained by summing up scores of each item of a certain sub-scale and then divide the total score by the number of items in that sub-scale. Higher scores correspond to greater psychological symptoms. For each sub-scale, a score higher than the mean score plus one deviation was considered as the cut-off point for higher risk of mental health problem (35) and coded as “1.” In comparison, lower scores were coded as “0.”

#### Independent Variable

Gender, age, education level, household register, marital status, living condition, employment status, occupation, and monthly household income per capita were included as demographic variables.

Status of chronic diseases and the BMI index were included as health-related factors. Participants were asked to state any history of chronic diseases, and their height and weight were self-reported to calculate the BMI index. Status of Chronic diseases was grouped as no chronic disease, one chronic disease and two or more chronic diseases. The BMI index was categorized as underweight (<18.5), normal (18.5–24) and overweight (≥24).

Environmental control variables included local community-level control measures and outbreak risk level of current location. Respondents were asked to report their current addresses at the district level and community-level control measures in their residential areas, which was classified as free entry/exit as usual, entry/exit control exercised, and lockdown. The outbreak risk level of respondents' current residential provinces was categorized into three levels according to cumulative confirmed COVID-19 cases, provinces with <100 cumulative confirmed cases, 100–1,000 cases, and over 1,000 cases were rated as low, medium, and high-risk areas, respectively.

Respondents were also asked to choose the source they obtained COVID-19 information from internet media platforms, traditional media like TV or newspaper, relatives or friends, and other sources.

### Statistical Analysis

Descriptive analysis was used to summarize the respondents' characteristics and psychological status. Frequencies (n) and percentage (%) were used to present categorical variables, while median and interquartile [M(Q)] and means and standard deviation (Mean ± SD) were used to present continuous variables. A univariate analysis (Chi-square test) was used to explore the significant associations between background variables and psychological symptoms. Multiple logistic regression models using scores of each sub-scale as outcome variables and statistically significant background variables as independent variables were built to identify factors associated with each of the five sub-scales. Odds ratios (OR) and the corresponding 95% confidence intervals (CIs) were reported. All the tests were two- sided, with a statistical significance level set at *p* < 0.05. Data were analyzed by IBM SPSS Version 19.0 (IBM Corp, 2010, Armonk, NY, USA).

### Ethics Statement

The protocol was approved by Ethics Committee of School of Public Health, Zhejiang University (approval number: ZGL202002-2). This study is anonymous and voluntary, and the respondents were informed that submission of the questionnaire implied informed consent. The data were kept confidential and the results did not identify the respondents personally.

## Results

### Study Sample Characteristics

Table 1 demonstrates the characteristics of the study sample. Of the 1,278 participants, over half of the sample were female (*n* = 707, 55.3%), and 725 (56.7%) participants were aged 70 and below. For education level, a total of 588 (46.0%) participants had attended primary school or below. 690 (54.0%) respondents had registered permanent residences in rural areas. In addition, approximately three-fourths were married or in a cohabitation situation (*n* = 954, 74.6%), and most of the participants (*n* = 903, 70.7%) lived with their spouses. The majority of the participants (*n* = 1,209, 94.6%) had retired from work. As for occupations, 608 (47.6%) participants were farmers, and only 33 (2.6%) participants worked in medical health institutions. Most (*n* = 852, 66.7%) of the respondents had an average household income between 600 and 6,000 yuan per month. A total of 514 (40.2%) participants had two or more chronic diseases, and 426 (33.3%) participants were overweight. Nearly half of the participants (*n* = 588, 46.0%) lived in areas with a medium-risk of COVID-19. The majority (*n* = 1,061, 83.0%) reported that an entry/exit control system was exercised in their community/village. A small proportion of the participants (*n* = 247, 19.3%) had obtained information about COVID-19 from Internet media platforms like WeChat. Traditional media channels, such as newspapers and television, remained the most widely used way to obtain information among the elderly (*n* = 572, 44.8%).

**Table 1:** Study sample characteristics (*N* = 1,278).

**Variables**	***n***	**%**
**Gender**		
Male	571	44.7
Female	707	55.3
**Age**		
≤ 70	725	56.7
70–80	459	35.9
>80	94	7.4
**Education**		
Primary school and below	588	46.0
Middle school	333	26.1
High school	202	15.8
Junior college	75	5.9
Bachelor's degree and above	80	6.3
**Residence**		
Urban	588	46.0
Rural	690	54.0
**Living condition**		
Living alone	122	9.5
Living with spouse	903	70.7
Living with children	232	18.2
Living in nursing house with others	21	1.6
**Marital status**		
Married/co-habited	954	74.6
Others	324	25.4
**Employment status**		
Retired	1,209	94.6
Employed	69	5.4
**Occupation**		
Health care worker	33	2.6
Civil servant	57	4.5
Workers in enterprises and institutions	458	35.8
Farmer	608	47.6
Others	122	9.5
**Household monthly income per person (Yuan)**		
<600	221	17.3
600–6,000	852	66.7
>6,000	205	16.0
**BMI^a^**		
Normal	744	58.2
Underweight	107	8.4
Overweight	426	33.3
**Chronic diseases**		
No chronic disease	264	20.7
One chronic disease	500	39.1
Two or more chronic diseases	514	40.2
**Outbreak risk level of current location^b^**		
Low	157	12.3
Medium	588	46.0
High	533	41.7
**Local community-level control measures**		
Free entry/exit as usual	34	2.7
Entry/exit control exercised	1,061	83.0
Lockdown	183	14.3
**Source of information**		
Internet media platforms	247	19.3
Newspaper and TV	572	44.8
Relatives or friends	406	31.8
Others	53	4.1

a *The BMI index has 1 missing value*.

b *According to authorized data from National Health Commission (accessed on March 20, 2020), the study classified 31 provinces (cities, autonomous regions) with cumulative confirmed cases <100, 100–999, and ≥1,000 as low, medium and high risk areas*.

### The Mental Health of the Participants During the Pandemic

As shown in Table 2, the participants reported relatively good mental health, with scores on most sub-scales of the PQEEPH <1. The median score on the fear scale reached 1.00, significantly higher than that of the other four scales (the Friedman test χ^2^ = 2,613.495, *p* < 0.001). Participants with higher risk of mental health problem on depression, neurasthenia, fear, anxiety, hypochondria were 13.8% (*n* = 177), 14.9% (*n* = 190), 14.7% (*n* = 188), 10.0% (*n* = 128), 11.9% (*n* = 152), respectively.

**Table 2:** Psychological status of Chinese elderly (*N* = 1,278).

**Psychological dimensions**	**Score range**	**Scores M (Q)**	**Scores (Mean ± SD)^a^**	**Higher risk of mental health problem, *n* (%)**
Depression	0–3	0.00 (0.33)	0.27 ± 0.48	177 (13.8)
Neurasthenia	0–3	0.00 (0.40)	0.28 ± 0.45	190 (14.9)
Fear	0–3	1.00 (0.83)	0.99 ± 0.59	188 (14.7)
Anxiety	0–3	0.00 (0.17)	0.18 ± 0.35	128 (10.0)
Hypochondria	0–3	0.00 (0.50)	0.24 ± 0.46	152 (11.9)

a *A score higher than mean score plus one deviation of each sub-scale is defined as the cut-off point*.

### Univariate Analysis of Participants' Characteristics With the Scores of the Five Sub-scales of the PQEEPH

As shown in Table 3, significant correlates of depression were marital status, education level, residence registration, employment status, occupation, monthly household income per capita, outbreak risk level of the current location, local community-level control measures, the BMI index, and chronic diseases (all *p* < 0.05). Significant correlates of anxiety were marital status, residence registration, living conditions, household monthly income per capita, local community-level control measures, source of COVID-19 information, the BMI index, and chronic diseases (all *p* < 0.05). Related factors of neurasthenia were residence registration, employment status, the BMI index, chronic diseases, and outbreak risk level of the current location (all *p* < 0.05). Correlates of fear and hypochondria were residence registration, employment status, the BMI index, and chronic diseases (all *p* < 0.05).

**Table 3:** Univariate analysis of participants' characteristics with the scores of the five sub-scales of the PQEEPH (*N* = 1,278)^a^.

**Variables**	**Depression (*N* = 177)**	**Neurasthenia (*N* = 190)**	**Fear (*N* = 188)**	**Anxiety (*N* = 128)**	**Hypochondria (*N* = 152)**
	***P*-value (χ^2^)**	***P*-value (χ^2^)**	***P*-value (χ^2^)**	***P*-value (χ^2^)**	***P*-value (χ^2^)**
Gender	0.423 (0.642)	0.419 (0.653)	0.153 (2.042)	0.276 (1.186)	0.717 (0.132)
Age	0.096 (4.691)	0.070 (5.323)	0.451 (1.593)	0.197 (3.251)	0.266 (2.647)
Education	**0.002** (17.458)	0.107 (7.621)	0.063 (8.910)	0.183 (6.222)	0.375 (4.237)
Residence	**0.003** (8.974)	**0.001** (10.375)	**0.001** (11.603)	**0.016** (5.809)	**<0.001** (15.811)
Living condition	0.061 (7.140)^d^	0.210 (4.469)^d^	0.742 (1.266)^d^	**0.016** (10.020)^d^	0.578 (1.899)^d^
Marital status	**0.024** (5.096)	0.050 (3.832)	0.808 (0.059)	**0.024** (5.106)	0.278 (1.178)
Employment status	**0.046** (3.964)	**0.012** (6.377)	**0.032** (4.619)	0.431 (0.621)	**0.006** (7.593)
Occupation	**0.004** (15.221)^d^	0.137 (6.889)^d^	0.251 (5.307)^d^	0.161 (6.417)^d^	0.156 (6.553)^d^
Household monthly income per person (Yuan)	**0.011** (9.064)	0.376 (1.955)	0.059 (5.655)	**0.004** (10.896)	0.265 (2.656)
BMI^b^	**0.010** (9.173)	**0.006** (10.217)	**0.002** (13.000)	**0.009** (9.339)	**0.042** (6.341)
Chronic diseases	**<0.001** (25.123)	**<0.001** (28.438)	**0.004** (10.930)	**<0.001** (26.086)	**<0.001** (19.157)
Outbreak risk level of current location^c^	**<0.001** (20.709)	**0.030** (6.981)	0.569 (1.129)	0.696 (0.724)	0.426 (1.707)
Local community-level control measure	0.051 (5.987)^d^	0.183 (3.399)	0.122 (4.210)	**0.018** (7.708)^d^	0.532 (1.239)^d^
Source of information	0.373 (3.121)	0.661 (1.592)	0.180 (4.894)	**0.033** (8.722)	0.509 (2.317)

a *Values were calculated from Chi-Square Test to examine the differences of scores of each sub-scale between populations with different characteristics. Significant P values are printed in bold. The complete univariate analysis form including different groups of all variables is attached in the Supplementary Table 1*.

b *The BMI index has 1 missing value*.

c *According to authorized data from National Health Commission (accessed on March 20, 2020), the study classified 31 provinces (cities, autonomous regions) with cumulative confirmed cases <100, 100–999, and ≥1,000 as low, medium and high risk areas*.

d *1 cell has expected count <5, the Fisher's exact test was used*.

### Factors Associated With Scores of the Five Sub-scales of the PQEEPH: A Multivariable Analysis

**Figure 1:**
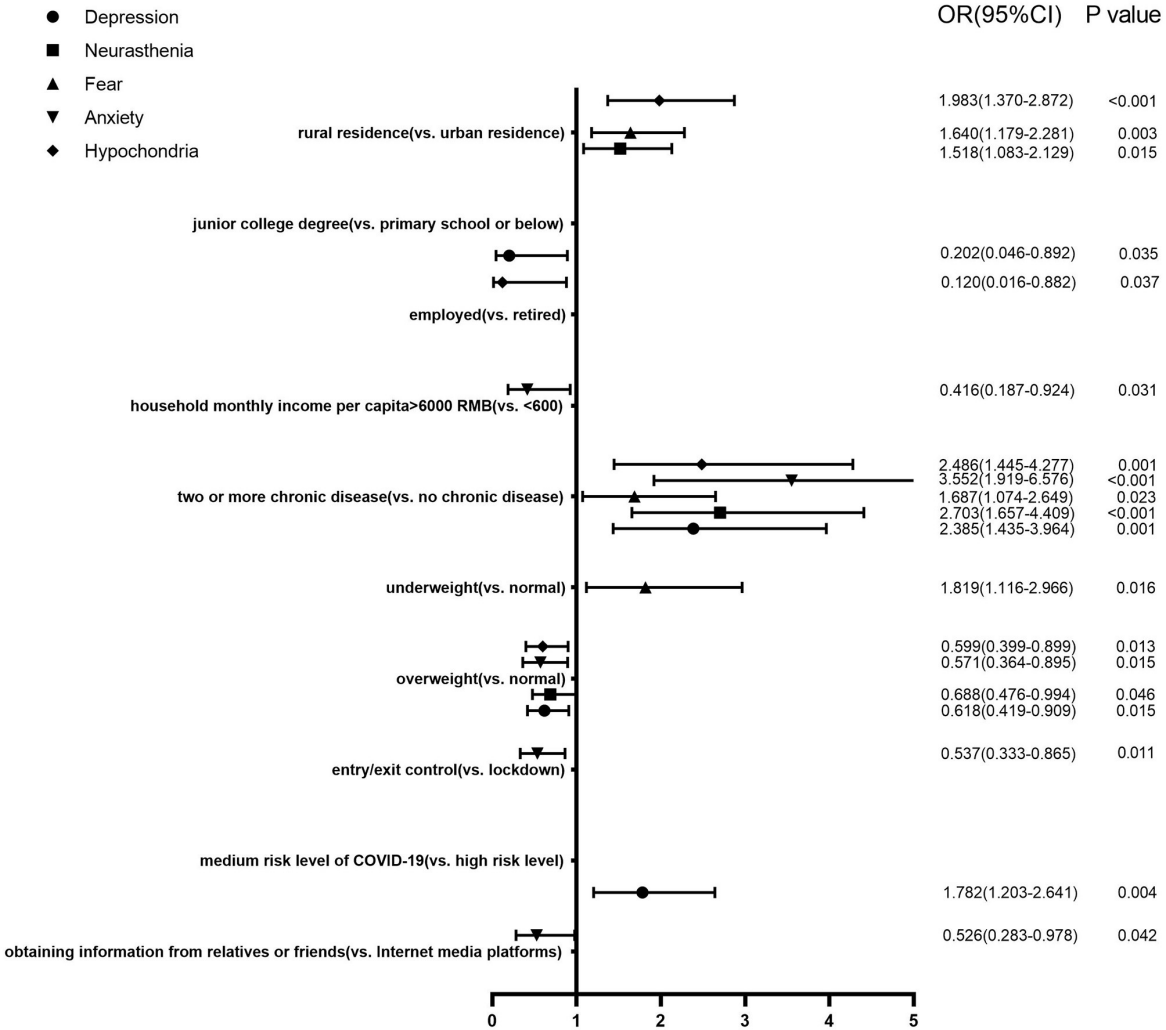
Factors associated with scores of the five sub-scales of the PQEEPH: a multivariable analysis (*N* = 1,278).

The multiple logistic regression models of scores of the five sub-scales of the PQEEPH (Figure 1) showed that health-related factors had the greatest impact on mental health. Respondents who had two or more chronic diseases were more likely to develop mental problems on all of the five sub-scales. As for the BMI index, those who were underweight experienced more fear, while being overweight was related to positive mental outcomes on the scale of depression, neurasthenia, anxiety, and hypochondria. Rural dwellers had higher levels of neurasthenia, fear, and hypochondria. The respondents with an education level of junior college (vs. primary school or below, OR = 0.202, 95%CI = 0.046–0.892) were less likely to be depressed, while those who lived in medium-risk regions (vs. high- risk region, OR = 1.782, 95%CI = 1.203–2.641) had higher risk of depression. An average household income higher than 6,000 Yuan per month (vs. <600 Yuan, OR = 0.416, 95%CI = 0.187–0.924), community-level entry/exit control (vs. cannot leave the house, OR = 0.537, 95%CI = 0.333–0.865), obtaining COVID-19 related information from relatives or friends (vs. Internet media platforms, OR = 0.526, 95%CI = 0.283–0.978) were protective factors against anxiety. Being employed (vs. retired, OR = 0.120, 95%CI = 0.016–0.882) helped reduce the risk of hypochondria.

## Discussion

The elderly in this study reported mild mental problems with median scores on all five sub-scales of the PQEEPH being ≤ 1, and significantly more symptoms of fear were reported. This finding was consistent with similar studies using the same instrument during SARS, human H7N9 avian influenza, and COVID-19 (35–37), indicating that fear is the most common and serious negative emotion during a public health emergency. More attention should, therefore, be paid to the recognition and mitigation of fear.

The logistic regression analyses showed that health-related factors had the greatest influence on mental status. Participants with two or more chronic diseases showed more symptoms on all of the five sub-scales of the PQEEPH. The elderly, especially those with underlying chronic diseases, are at higher risk of contracting COVID-19 and could suffer from more severe and even fatal symptoms once infected (38). Additionally, people with chronic diseases could have difficulty obtaining maintenance treatments during COVID-19 due to strict epidemic control measures. More efforts should be done to promote the elderly with chronic diseases to uptake prevention behaviors, meanwhile, convenient and safe way of obtaining medical treatments during the epidemic should be provided for the elderly in need. Consistent with previous studies (39–41), underweight people reported poorer mental health. Overweight, however, was unexpectedly related to better mental states on four sub-scales. Lockdown during the pandemic has confined people to very limited spaces and impeded participation of physical exercise (42), but overweight people could be psychologically less affected as they participated less in physical activities (43). Another possible explanation is that overweight people had a poorer health awareness (44) and thereby suffered less psychological burden of the epidemic.

Several demographic factors, including residence registration, education level, employment status, and household monthly income per capita, were found to influence the five dimensions of the PQEEPH differently. Compared with those living in urban areas, rural dwellers expressed more neurasthenia, fear, and hypochondria. Although urban areas had more confirmed cases, medical resources and sanitary conditions in cities are better than in towns and villages, which increases the chances of surviving the virus (45). Besides, cities have better educational resources, and greater efforts have been made to publicize knowledge of epidemic prevention measures, which could alleviate the public's negative emotions (46). Therefore, COVID-19 related health education programs should be strengthened among rural dwellers to mitigate risk of infection. Adequate epidemic protective equipment should also be guaranteed for them and health care systems in rural areas should be further enhanced in the long term.

Participants with a junior college education level were at significantly lower risk of getting depressed than those who only attended primary school and below, which is similar with previous findings (24, 47). The elderly with a lower degree of education were less capable of getting access to and comprehending accurate information and facts regarding COVID-19, such as hygiene and precautionary measures. Health education on COVID-19 should thus pay more attention to this group using more acceptable forms like pictures or videos (48). Although those who were still at work may be at higher risk of exposure to the virus, being employed was surprisingly found to be associated with lower level of hypochondria. Actually, to prevent the spread of the virus, only those factories which passed strict examinations can be approved to reopen (49), therefore the contact with colleagues at work may not trigger panic. Besides, those who were still at work can be less likely to develop hypochondria symptoms as they could have better physical health (50) and working can help provide distraction from various worries (51), the mediation analysis also showed that the influence of employment status on hypochondria symptoms was partially mediated by health status (indirect effect = −0.063, *p* < 0.001) (see Supplementary Table 2). In line with previous studies (45, 52), the elderly with low average household incomes experienced more anxiety. Special social security measures could be established to help fight the economic challenges.

Other than demographic and health-related factors, environmental control variables were also significantly associated with certain sub-scale. Consistent with previous studies reporting that epidemic control measures like quarantine can have serious impacts on mental health (11, 53, 54), the elderly in our study who could not leave their house showed higher levels of anxiety than those who lived in communities where entry/exit controls were exercised. Against the backdrop of lockdown, offline social activities were inaccessible due to strict epidemic control measures (55, 56), while online interaction could also hardly be utilized by the elderly due to limited access and literacy in digital resources (57). The significantly reduced contact with family members and friends generated social and psychological isolation, triggering psychiatric disorders. Besides, the difficulty in obtaining basic living needs during lockdown could further exacerbate older adults' negative emotions of anxiety (15). However, it should also be noted that epidemic control measures play an important role in containing infectious diseases (58). Those in communities with no control measures also did not show a significantly lower level of anxiety. Therefore, community-level control measures are still necessary before the epidemic comes to the end, and close attention should be paid to the psychological state of those under strict epidemic control measures at the same time to recognize and address psychological problems in time. In contrast to previous studies reporting that people in most affected areas had significantly more negative emotions (9, 52), the elderly in our study who lived in regions with medium- risk levels of COVID-19 showed higher level of depression than those in high-risk regions. Those in high- risk areas were more motivated and had more access to gain knowledge on COVID-19, and a better understanding of the epidemic status can reduce the risk of developing negative emotions (59). High quality medical resources, strict and effective prevention measures as well as the strengthened public health systems in high- risk regions can also help alleviate psychological distress levels (16). Less than one-fifth of our participants obtained COVID-19 related information from Internet media platforms, and they expressed more anxiety than those who acquired information on COVID-19 from relatives or friends. There exist disparities in older adults' access to and knowledge on advanced technologies, and it would also be more difficult for them to distinguish accurate facts from fake information. Instead, family members are the core component of the social support network for the elderly (60). Apart from informing the elderly of important messages on COVID-19, relatives also conveyed spiritual comfort for the elderly, mitigating their anxiety over the pandemic. Therefore, there is a need to enhance family support to outreach COVID-19 information and promote preventive measures for the elderly during the epidemic.

Several limitations of this study should be stated. First, these findings' generalizability is limited, as the online survey only included those who had access to the Internet. However, online surveys can be the most appropriate method for data collection during an epidemic since it can avoid transmission and the distribution of the survey offline was not feasible under strict epidemic control measures. Second, this study's cross-sectional design prevented us from drawing causal associations. Third, the self-reported psychological status may not always be aligned with professional assessment, and recall and social desirability bias may also exist. Besides, despite that the PQEEPH can better reveal epidemic-specific emotions, comparison between older adults' mental health status in our study and that during non-epidemic periods can be restricted as the scale is epidemic-specific. While during epidemics, little attention has been paid to mental health among the elderly. Moreover, some important factors, such as contact history, relatives/friends who have contracted COVID-19, social support, and existing mental health problems, should be included in future studies. Notwithstanding all the above limitations, this study is one of the few studies investigating the psychological status of older adults in China during COVID-19. A large number of respondents from all the provinces in mainland China were recruited, enabling us to obtain a wide range of participants with various demographic backgrounds. Therefore, these findings provide important information for developing psychological interventions to alleviate negative emotions among the elderly during the outbreak of COVID-19.

## Conclusion

The elderly, who are at higher risk of getting affected both physically and psychologically during the COVID-19, deserve particular attention from local governments, health authorities as well as the civil society. This study explored Chinese elderly residents' mental health status during COVID-19 and relevant factors. The elderly in our study had mild degree of negative emotions but reported significantly more fear symptoms, suggesting that psychological interventions should give more attention to recognize and alleviate fear. Health condition showed the greatest impact on mental health, more attention should thus be paid to older adults with poorer physical health. Higher education level, better economic condition, and obtaining COVID-19 information from friends or relatives were associated with lower risk of mental health problems, while rural dwellers and those lived in medium risk or lockdown regions experienced significantly more symptoms. These findings can be used to formulate psychological interventions to improve the mental health and psychological resilience of the elderly during the COVID-19 epidemic.

## Data Availability Statement

The raw data supporting the conclusions of this article will be made available by the authors, without undue reservation.

## Author Contributions

HW and ZC conceived and designed the study. YC and BC assisted in questionnaire design and data collection. HC, YL, and HZ assisted in data collection. RZ and HC contributed to the statistical analysis and drafted the manuscript. LZ participated in statistical analysis. HW, RZ, and HC finalized the manuscript. All authors have read and agreed to the final version of the manuscript.

## Conflict of Interest

The authors declare that the research was conducted in the absence of any commercial or financial relationships that could be construed as a potential conflict of interest.
