# Supplementary material for: Mental Health Status of the Elderly Chinese Population During COVID-19: An Online Cross-Sectional Study
Source: Front Psychiatry. 2021 May 12;12:645938. doi: 10.3389/fpsyt.2021.645938 (PMC8149938; doi:10.3389/fpsyt.2021.645938)
Supplement: Supplementary file 1 [file Table_1.docx]

**Table S1.** Univariate analysis of participants’ characteristics with the scores of the five sub-scales of the PQEEPH (N=1278)^a^.

| **Variables** | **Depression**  (N=177) | **Neurasthenia**  (N=190) | **Fear**  (N=188) | **Anxiety**  (N=128) | **Hypochondria**  (N=152) |
| --- | --- | --- | --- | --- | --- |
| **Gender** |  |  |  |  |  |
| Male | 84(14.7) | 90(15.8) | 75(13.1) | 63(11.0) | 70(12.3) |
| Female | 93(13.2) | 100(14.1) | 113(16.0) | 65(9.2) | 82(11.6) |
| χ^2^ | 0.642 | 0.653 | 2.042 | 1.186 | 0.132 |
| *P* | 0.423 | 0.419 | 0.153 | 0.276 | 0.717 |
| **Age** |  |  |  |  |  |
| <=70 | 96(13.2) | 98(13.5) | 104(14.3) | 66(9.1) | 85(11.7) |
| 70-80 | 61(13.3) | 71(15.5) | 66(14.4) | 48(10.5) | 51(11.1) |
| >80 | 20(21.3) | 21(22.3) | 18(19.1) | 14(14.9) | 16(17.0) |
| χ^2^ | 4.691 | 5.323 | 1.593 | 3.251 | 2.647 |
| *P* | 0.096 | 0.070 | 0.451 | 0.197 | 0.266 |
| **Education** |  |  |  |  |  |
| Primary school  and below | 98(16.7)^i^ | 103(17.5) | 92(15.6) | 70(11.9) | 81(13.8) |
| Middle school | 51(15.3)^i^ | 43(12.9) | 57(17.1) | 33(9.9) | 37(11.1) |
| High school | 19(9.4)^i,ii^ | 29(14.4) | 27(13.4) | 15(7.4) | 19(9.4) |
| Junior college | 2(2.7)^ii^ | 7(9.3) | 4(5.3) | 4(5.3) | 8(10.7) |
| Bachelor’s degree and above | 7(8.8)^i,ii^ | 8(10.0) | 8(10.0) | 6(7.5) | 7(8.8) |
| χ^2^ | 17.458 | 7.621 | 8.910 | 6.222 | 4.237 |
| *P* | **0.002** | 0.107 | 0.063 | 0.183 | 0.375 |
| **Residence** |  |  |  |  |  |
| Urban | 63 (10.7) | 67(11.4) | 65(11.1) | 46(7.8) | 47(8.0) |
| Rural | 114(16.5) | 123(17.8) | 123(17.8) | 82(11.9) | 105(15.2) |
| χ^2^ | 8.974 | 10.375 | 11.603 | 5.809 | 15.811 |
| *P* | **0.003** | **0.001** | **0.001** | **0.016** | **<0.001** |
| **Living condition** |  |  |  |  |  |
| Living alone | 17(13.9) | 25(20.5) | 17(13.9) | 14(11.5)^i,ii^ | 17(13.9) |
| Living with spouse | 113(12.5) | 125(13.8) | 129(14.3) | 77(8.5)^ii^ | 101(11.2) |
| Living with children | 45(19.4) | 38(16.4) | 38(16.4) | 36(15.5)^i^ | 32(13.8) |
| Living in nursing house with others | 2(9.5) | 2(9.5) | 4(19.0) | 1(4.8)^i,ii^ | 2(9.5) |
| χ^2^ | 7.140^d^ | 4.469^d^ | 1.266^d^ | 10.020^d^ | 1.899^d^ |
| *P* | 0.061 | 0.210 | 0.742 | **0.016** | 0.578 |
| **Marital status** |  |  |  |  |  |
| Married/Co-habited | 120(12.6) | 131(13.7) | 139(14.6) | 85(8.9) | 108(11.3) |
| Others | 57(17.6) | 59(18.2) | 49(15.1) | 43(13.3) | 44(13.6) |
| χ^2^ | 5.096 | 3.832 | 0.059 | 5.106 | 1.178 |
| *P* | **0.024** | 0.050 | 0.808 | **0.024** | 0.278 |
| **Employment status** | | | | | |
| Retired | 173(14.3) | 187(15.5) | 184(15.2) | 123(10.2) | 151(12.5) |
| Employed | 4(5.8) | 3(4.3) | 4(5.8) | 5(7.2) | 1(1.4) |
| χ^2^ | 3.964 | 6.377 | 4.619 | 0.621 | 7.593 |
| *P* | **0.046** | **0.012** | **0.032** | 0.431 | **0.006** |
| **Occupation** |  |  |  |  |  |
| Health care worker | 6(18.2)^i,ii^ | 6(18.2) | 4(12.1) | 4(12.1) | 4(12.1) |
| Civil servant | 6(10.5)^i,ii^ | 4 (7.0) | 6(10.5) | 3 (5.3) | 6(10.5) |
| Workers in enterprises and institutions | 50(10.9)^ii^ | 60(13.1) | 57(12.4) | 46(10.0) | 49(10.7) |
| Farmer | 106(17.4)^i^ | 104(17.1) | 104(17.1) | 69(11.3) | 85(14.0) |
| Others | 9(7.4)^i,ii^ | 16(13.1) | 17(13.9) | 6(4.9) | 8(6.6) |
| χ^2^ | 15.221^d^ | 6.889^d^ | 5.307^d^ | 6.417^d^ | 6.553^d^ |
| *P* | **0.004** | 0.137 | 0.251 | 0.161 | 0.156 |
| **Household monthly income per person (Yuan)** | | | | | |
| <600 | 36(16.3)^i^ | 35(15.8) | 39(17.6) | 32(14.5)^i^ | 23(10.4) |
| 600-6000 | 126(14.8)^i^ | 131(15.4) | 129(15.1) | 86(10.1)^i,ii^ | 110(12.9) |
| >6000 | 15(7.3)^ii^ | 24 (11.7) | 20(9.8) | 10(4.9)^ii^ | 19(9.3) |
| χ^2^ | 9.064 | 1.955 | 5.655 | 10.896 | 2.656 |
| *P* | **0.011** | 0.376 | 0.059 | **0.004** | 0.265 |
| **BMI^b^** |  |  |  |  |  |
| Normal | 111(14.9)^i,ii^ | 116(15.6) ^i,ii^ | 112(15.1)^i^ | 79(10.6)^i,ii^ | 100(13.4)^i^ |
| Underweight | 22(20.6) ^ii^ | 25(23.4) ^ii^ | 27(25.2)^ii^ | 18(16.8)^ii^ | 15(14)^i,ii^ |
| Overweight | 44(10.3) ^i^ | 49(11.5) ^i^ | 49(11.5)^i^ | 31(7.3)^i^ | 37(8.7)^ii^ |
| χ^2^ | 9.173 | 10.217 | 13.000 | 9.339 | 6.341 |
| *P* | **0.010** | **0.006** | **0.002** | **0.009** | **0.042** |
| **Chronic diseases** |  |  |  |  |  |
| No chronic disease | 22(8.3) ^i^ | 23(8.7) ^i^ | 30(11.4)^i^ | 14(5.3)^i^ | 18(6.8) ^i^ |
| One chronic disease | 54(10.8) ^i^ | 58(11.6) ^i^ | 62(12.4)^i^ | 36(7.2)^i^ | 49(9.8) ^i^ |
| Two or more chronic diseases | 101(19.6) ^ii^ | 109(21.2) ^ii^ | 96(18.7)^ii^ | 78(15.2)^ii^ | 85(16.5) ^ii^ |
| χ^2^ | 25.123 | 28.438 | 10.930 | 26.086 | 19.157 |
| *P* | **<0.001** | **<0.001** | **0.004** | **<0.001** | **<0.001** |
| **Outbreak risk level of current location^c^** | | | | | |
| Low | 23(14.6)^i,ii^ | 16(10.2)^i^ | 19 (12.1) | 16(10.2) | 14 (8.9) |
| Medium | 107 (18.2)^ii^ | 103(17.5)^i^ | 91(15.5) | 63 (10.7) | 70 (11.9) |
| High | 47(8.8)^i^ | 71 (13.3)^i^ | 78(14.6) | 49(9.2) | 68 (12.8) |
| χ^2^ | 20.709 | 6.981 | 1.129 | 0.724 | 1.707 |
| *P* | **<0.001** | **0.030** | 0.569 | 0.696 | 0.426 |
| **Local community-level control measure** | | | | | |
| Free entry/exit as usual | 5(14.7) | 6(17.6) | 5(14.7) | 5(14.7)^i^ | 3(8.8) |
| Entry/exit control exercised | 136(12.8) | 149(14.0) | 147(13.9) | 95(9.0)^ii^ | 123(11.6) |
| Lockdown | 36(19.7) | 35(19.1) | 36(19.7) | 28(15.3)^i,ii^ | 26(14.2) |
| χ^2^ | 5.987^d^ | 3.399 | 4.210 | 7.708^d^ | 1.239^d^ |
| *P* | 0.051 | 0.183 | 0.122 | **0.018** | 0.532 |
| **Source of information** | | | | | |
| Internet media platforms | 26(10.5) | 32(13.0) | 41(16.6) | 21(8.5)^i^ | 32(13.0) |
| Newspaper and TV | 84(14.7) | 89(15.6) | 71(12.4) | 72(12.6)^i^ | 67(11.7) |
| Relatives or friends | 58(14.3) | 63(15.5) | 69(17.0) | 33(8.1)^i^ | 50(12.3) |
| Others | 9(17.0) | 6(11.3) | 7(13.2) | 2(3.8)^i^ | 3(5.7) |
| χ^2^ | 3.121 | 1.592 | 4.894 | 8.722 | 2.317 |
| *P* | 0.373 | 0.661 | 0.180 | **0.033** | 0.509 |

^a^ Values were calculated from Chi-Square Test to examine the differences of scores of each sub-scale between populations with different characteristics. For categories of variables with significant χ^2^ results (Significant at *P*<.05 between the groups), multiple comparisons between each 2 categories are done by Bonferroni test. Within each column, when two means share same superscript (i, ii), it indicates that they were not statistically different (*P*>.05) from one another.

^b^ The BMI index has 1 missing value.

^c^ According to authorized data from National Health Commission (accessed on March 20, 2020), the study classified 31 provinces (cities, autonomous regions) with cumulative confirmed cases <100, 100-999, and ≥1000 as low, medium and high risk areas.

^d^ 1 cell has expected count less than 5, the Fisher’s exact test was used.
