# Supplementary material for: Mental Health Status of the Elderly Chinese Population During COVID-19: An Online Cross-Sectional Study
Source: Front Psychiatry. 2021 May 12;12:645938. doi: 10.3389/fpsyt.2021.645938 (PMC8149938; doi:10.3389/fpsyt.2021.645938)
Supplement: Supplementary file 2 [file Table_2.docx]

**Table S2.** Estimates of direct, indirect standardized effects for the structural equation model (chronic diseases as the mediating variable)

| **Effect** | **β** | **P-value** |
| --- | --- | --- |
| **Direct effects** |  |  |
| Employment status→ chronic diseases | -0.517 | <0.001 |
| Chronic diseases→ hypochondria | 0.122 | <0.001 |
| Employment status→ hypochondria | -0.261 | 0.001 |
| **Indirect effects** |  |  |
| Employment status→ chronic diseases→ hypochondria | -0.063 | <0.001 |
